# An open-label clinical trial to investigate the efficacy and safety of corifollitropin alfa combined with hCG in adult men with hypogonadotropic hypogonadism

**DOI:** 10.1186/s12958-017-0232-y

**Published:** 2017-03-07

**Authors:** Eberhard Nieschlag, Pierre-Marc G. Bouloux, Barbara J. Stegmann, R. Ravi Shankar, Yanfen Guan, Anjela Tzontcheva, Christine McCrary Sisk, Hermann M. Behre

**Affiliations:** 1 University Hospital of Muenster, Center of Reproductive Medicine and Andrology, Domagkstraße 11, D-48149 Muenster, Germany; 2 The Royal Free Hospital, Pond St, London, NW3 2QG UK; 3 Merck & Co., Inc., 2000 Galloping Hill Road, Kenilworth, NJ 07033 USA; 4 University Hospital Halle (Saale), Martin Luther University Halle-Wittenberg, Ernst-Grube-Str. 40, D-06120 Halle, Germany

**Keywords:** Corifollitropin alfa, Gonadotropin deficiency, Hypogonadotropic hypogonadism, Male infertility, Testis

## Abstract

**Background:**

Hypogonadotropic hypogonadism (HH) in men results in insufficient testicular function and deficiencies in testosterone and spermatogenesis. Combinations of human chorionic gonadotropin (hCG) and recombinant follicle-stimulating hormone (recFSH) have been successful in the treatment of HH. Corifollitropin alfa is a long-acting FSH-analog with demonstrated action in women seeking infertility care. The aim of this study was to investigate the efficacy and safety of corifollitropin alfa combined with hCG to increase testicular volume and induce spermatogenesis in men with HH.

**Methods:**

This was a Phase III, multi-center, open-label, single-arm trial of corifollitropin alfa in azoospermic men aged 18 to 50 years with HH. After 16 weeks of pretreatment of 23 subjects with hCG alone, 18 subjects with normalized testosterone (T) levels who remained azoospermic entered the 52-week combined treatment phase with hCG twice-weekly and 150 μg corifollitropin alfa every other week. The increase in testicular volume (primary efficacy endpoint) and induction of spermatogenesis resulting in a sperm count ≥1 × 10^6^/mL (key secondary efficacy endpoint) during 52 weeks of combined treatment were assessed. Safety was evaluated by the presence of anti-corifollitropin alfa antibodies and the occurrence of adverse events (AEs).

**Results:**

Mean (±SD) testicular volume increased from 8.6 (±6.09) mL to 17.8 (±8.93) mL (geometric mean fold increase, 2.30 [95% CI: 2.03, 2.62]); 14 (77.8%) subjects reached a sperm count ≥1 × 10^6^/mL. No subject developed confirmed anti-corifollitropin alfa antibodies during the trial. Treatment was generally well tolerated.

**Conclusions:**

Corifollitropin alfa 150 μg administrated every other week combined with twice-weekly hCG for 52 weeks increased testicular volume significantly, and induced spermatogenesis in >75% of men with HH who had remained azoospermic after hCG treatment alone.

**Trial registration:**

ClinicalTrials.gov: NCT01709331.

**Electronic supplementary material:**

The online version of this article (doi:10.1186/s12958-017-0232-y) contains supplementary material, which is available to authorized users.

## Background

Male hypogonadotropic hypogonadism (HH) is characterized by impairment in the secretion of the pituitary gonadotropins, follicle-stimulating hormone (FSH), and luteinizing hormone (LH), resulting in insufficient testicular function and deficiencies in testosterone (T) and spermatogenesis [1, 2]. The onset of HH prior to adolescence results in delayed or absent puberty with the respective clinical consequences, whereas onset in adult life leads to hypogonadism with loss of androgenicity and infertility [1, 2]. The approach to treatment of HH in males varies based on the desired outcome; T or human chorionic gonadotropin (hCG) therapy alone are sufficient to induce virilization. However, treatment with gonadotropins (LH and FSH) or gonadotropin-releasing hormone (GnRH) is required to improve testicular function and induce spermatogenesis [1, 2]. The hormone hCG has been successfully used to replace LH-activity since the 1950s. For FSH-activity, human menopausal gonadotropin (hMG) became available in the 1960s [3, 4] before recombinant FSH was synthesized in the 1990s and became the standard therapy for replacement in HH patients for stimulation of spermatogenesis [5]. In a previous clinical trial of recombinant FSH (recFSH; Puregon®) in men with HH (*N* = 30), hCG in combination with recFSH resulted in a doubling of the combined testicular volume from 11.4 to 24.0 mL, and achievement of sperm counts ≥1 × 10^6^ in 14 of 30 (47%) men who had remained azoospermic after T levels had been normalized during 16 weeks of pretreatment with hCG alone [6]. However, due to the short half-life of FSH, recFSH needs to be injected 3 times per week for extended periods of several months to years.

Corifollitropin alfa (MK-8962; formerly Org 36286/SCH 900962) is a recombinant gonadotropin consisting of the α-subunit of human FSH and a hybrid subunit composed of the sequence of the β-subunit of human FSH and the carboxy-terminal peptide part of the β-subunit of hCG. In women seeking infertility care, treatment with corifollitropin alfa produced a similar therapeutic response compared with that of recFSH [7–9]. Corifollitropin alfa has the same pharmacodynamic (PD) profile as recFSH, but an approximately two-fold longer elimination half-life (t_1/2_) and an almost four-fold extended time interval to peak serum levels relative to that of recFSH [10]. As a result, a single injection of corifollitropin alfa replaces 7 days of daily recFSH. Since treatment of HH in males usually requires long-term treatment with frequent injections of recFSH, the use of corifollitropin alfa, requiring fewer injections, may result in fewer medication errors and improved compliance.

The majority of clinical trials comparing corifollitropin alfa with daily recFSH have been conducted in women undergoing controlled ovarian stimulation for the development of multiple follicles and pregnancy for assisted reproductive technology (ART). A Phase 1, open-label trial with corifollitropin alfa conducted in 13 men with HH demonstrated that a single dose of 15 μg corifollitropin alfa was able to induce an increase in serum inhibin-B concentrations [11]. The objective of the present study was to investigate the efficacy and safety of corifollitropin alfa in combination with hCG to increase testicular growth and induce spermatogenesis resulting in a sperm count ≥1 × 10^6^/mL in adult men with HH who remained azoospermic after treatment with hCG alone.

## Methods

### Study design and population

This was a phase III, multi-center, open label, single-arm trial conducted in 13 centers in Australia, Germany, Italy, Poland, Spain, and the United Kingdom from February 14, 2013 to April 8, 2015 (Protocol: MK-8962-031-01, formerly known as SCH 900962, P07937; ClinicalTrials.gov: NCT01709331). The study protocol was approved by the institutional review boards at every study center and informed consent was obtained before the initiation of any study procedures. The study was conducted in accordance with the principles of Good Clinical Practice and applicable country and/or local statutes and regulations regarding ethical committee review, informed consent, and the protection of human subjects participating in biomedical research.

The study design is illustrated in Additional file 1: Figure S1. The trial began at Week -16 with an hCG-only pretreatment phase, during which eligible men with HH were treated with 1500 IU of hCG (Merck & Co., Inc., Kenilworth, NJ, USA) administered subcutaneously (SC) twice-weekly for 16 weeks to normalize T levels and to determine if hCG alone would be sufficient to achieve spermatogenesis. The dose could be increased to 3000 IU twice-weekly after 8 weeks if the Total T level remained below the unequivocal lower limits of the normal range (8.68 nmol/L [250 ng/dL]). Continuation into the 52-week combined treatment phase (corifollitropin alfa with hCG) was limited to subjects with normalized T levels who remained azoospermic at the conclusion of the pretreatment phase.

Day 1 of the trial was the first day of the 52-week combined treatment phase (corifollitropin alfa with hCG). During this phase, subjects received corifollitropin alfa (150 μg SC) once every two weeks in addition to twice-weekly hCG. The initial hCG dose in the combined treatment phase was the same as the final dose in the pretreatment phase; the dose was then adjusted up to 3000 IU or down to 1500 IU as needed to maintain Total T and estradiol (E2) levels within acceptable ranges according to the investigator’s discretion. hCG was administered on the same two days every week (Monday/Thursday or Tuesday/Friday), and corifollitropin alfa was given on one of the days when hCG was administered. The final follow-up visit occurred at least 21 days after the last dose of corifollitropin alfa and at least seven days after the last dose of hCG. At this visit, all subjects were assessed for any adverse events (AEs) that occurred after the administration of the last dose of trial medication. Blood samples were collected for the last assessment of hormones and anti-drug antibodies. Information was also collected on any treatment the subjects received, for either AEs or for the treatment of HH, after the trial.

Men between 18 and 50 years, with congenital or acquired HH, who had azoospermia, low circulating levels of gonadotropins (FSH and LH ≤2 IU/L) and T (≤6 nmol/L), presence of scrotal testes, adequate replacement of other pituitary hormones (if applicable), good general physical and mental health, and ability and willingness to comply with the protocol and provide written informed consent were included in the study. Important exclusion criteria included the presence of primary hypogonadism (e.g., Klinefelter syndrome), history of unilateral or bilateral cryptorchidism, and history or presence of testicular pathology of clinical importance and/or vasectomy. Men who were treated with FSH, hCG, or GnRH within the previous 3 months or for >1 month within the previous 6 months before signing informed consent were excluded. Other exclusion criteria were hypophysectomy within 6 months prior to screening, past or present oncologic treatment, diabetes mellitus, untreated hyperprolactinemia, uncontrolled non-gonadal endocrinopathies (thyroid, adrenal, pituitary disorders), history of alcohol or drug abuse, and administration of hormonal preparations, agents known to impair testicular function or affect sex hormone secretion, and known or suspected teratogens within 1 month before the start of screening. Prior use of androgen preparations was permitted if the patient underwent the wash-out period to bring the total T down to ≤6 nmol/L (173 ng/dL).

### Endpoints

The primary efficacy endpoint was the change from Day 1 to Week 52 (i.e., during the combined treatment phase) in log-transformed testicular volume (measured as the sum of ultrasound determination of volumes of left and right testes). The key secondary efficacy endpoint was the percentage of subjects with induced spermatogenesis resulting in a sperm count ≥1 × 10^6^/mL at any time point at or before Week 52. Other secondary efficacy endpoints included serum concentrations of the hormones T, E2, FSH, sex hormone-binding globulin (SHBG), inhibin B, anti-Müllerian hormone (AMH), and luteinizing hormone (LH) measured during the combined treatment phase. The primary safety endpoint was the development of anti-corifollitropin alfa antibodies.

### Efficacy assessments

#### Determination of testicular volume and semen analysis

Testicular ultrasound was performed to measure and record the maximum longitudinal, antero-posterior, and transverse diameters, and evaluate the sonographic pattern of each testis at weeks -16, -8, and -1 of the hCG pretreatment phase, the first day of administration of corifollitropin alfa, every 4 weeks between weeks 4 to 52, and at discontinuation if the subject in the combined treatment phase withdrew before week 52.

Semen samples (produced by masturbation after sexual abstinence for at least 48 h) at screening, week -1 (last week of the hCG pretreatment phase and prior to start of the combined treatment phase), weeks 16, 28, 40, and 52, and discontinuation in subjects who withdrew prior to week 52, were analyzed according to the WHO manual (2010) [12]. Investigational sites received training for the standard operating procedures of semen analysis prior to the start of study. Analyses for semen volume, sperm density, motility, and morphology were performed at local semen laboratories using standardized methodology across all study sites. Azoospermia was diagnosed if no spermatozoa were found in the sediment of a centrifuged sample.

#### Endocrine parameters

Blood samples were collected before the injection(s) of hCG or corifollitropin alfa for the measurement of T, E2, FSH, SHBG, inhibin B, AMH, and LH at weeks -16, -8, and -1 of the hCG pretreatment phase, the first day of administration of corifollitropin alfa, every 4 weeks between weeks 4 to 52, at discontinuation if the subject in the combined treatment phase withdrew before week 52, and at the follow-up visit. Total T and E2 concentrations were determined centrally by Quest, Valencia, CA, USA, using liquid chromatography–mass spectrometry (LCMS). Serum FSH, SHBG, and LH concentrations were determined centrally by Analytical Biochemical Laboratory (ABL), Assen, The Netherlands, using kits for time-resolved fluoroimmunoassays (AutoDelfia, Wallac Oy, Finland). Inhibin B and AMH serum concentrations were analyzed centrally by ABL, Assen, The Netherlands, using a validated enzyme immunoassay (EIA) kit (Gen II ELISA; Beckman Coulter, Chaska, MN, USA).

### Safety assessments

Adverse events (AEs) and serious AEs (SAEs) were assessed throughout the study. Routine blood chemistry, hematology, urinalysis, physical and andrological examinations, and body weight, height, blood pressure, and heart rate measurements were performed throughout the study. Development of anti-corifollitropin alfa antibodies was a primary safety endpoint. Serum samples were collected for the assessment of anti-corifollitropin alfa antibodies at weeks -16 and -1 of the hCG pretreatment phase, weeks 4, 16, 28, 40, and 52, at discontinuation if the subject in the combined treatment phase withdrew prior to week 52, and at the follow-up visit. All blood sampling for antibodies was performed prior to the scheduled injection(s) of hCG or corifollitropin alfa for that day. Other events of clinical interest included elevated aspartate aminotransferase (AST) or alanine aminotransferase (ALT) ≥3 × the upper limit of normal (ULN) and elevated total bilirubin ≥2 × ULN and simultaneous alkaline phosphatase <2 × ULN and hypersensitivity reactions (skin rash, urticaria, hypotension, allergic asthma, chest tightness, bronchospasm, dyspnea, or wheezing following injection with corifollitropin alfa).

### Statistical analyses

It was initially planned that 40 to 50 subjects would be screened. Assuming a screen failure rate of 50 to 60%, this would result in about 20 subjects that were expected to enter the hCG pretreatment phase. A minimum of 10 subjects with normalized T levels who remained azoospermic at the end of the pretreatment phase were expected to enter the combined treatment phase. Sample size estimates were based on the results from a previous trial with recFSH in HH men [6]. The geometric mean increase in testicular volume during 48 weeks of recFSH treatment was 1.77 or, equivalently, 0.57 on log-scale (base e = 2.178) with a standard deviation (SD) of 0.36 (*n* = 16). Under the assumptions of a delta of 0.55 and a SD of 0.4 (both on the log-scale) and a one-sided significance level alpha of 0.025, a total of 10 azoospermic men (with normalized T levels after 16 weeks of hCG pretreatment) are sufficient for a 90% power to statistically demonstrate an increase of testicular volume following the addition of corifollitropin alfa to hCG treatment. In addition, when either the increase from Day 1 is somewhat smaller (e.g., 0.45 on log-scale) or the variation is somewhat larger (e.g., 0.5 on log-scale), the power is close to 90%.

Efficacy analyses were based on the Full Analysis Set population, including all subjects who received any dose of corifollitropin alfa and who had a baseline and at least one post-baseline measurement of the efficacy variable in question. The mean change from Day 1 (first day of the combined treatment phase) in log-transformed testicular volume was analyzed using a mixed model with a fixed-effect for time point and a random effect for subject. The mixed model accounted for missing data, assuming that data were missing at random. In addition, Last Observation Carried Forward approaches were performed for sensitivity analyses. For each time point (i.e., Weeks 4 to 52), the mean change from Day 1 to that time point and the associated 95% CI were calculated. The geometric mean increase in testicular volume and its 95% CI were obtained by exponentiation. For secondary efficacy endpoints, summary statistics included the number of observations, mean, SD, median, minimum, and maximum for continuous variables; frequency distributions were provided for categorical variables. For endocrine parameters, values below the lower limit of quantitation (LLOQ) were set to 0.5 × LLOQ for the calculation of descriptive statistics. For each parameter, descriptive statistics were calculated per assessment, including the ‘last measurement’ assessment and the minimum and maximum post-baseline value. The calculations were also performed for the absolute and percentage changes from baseline.

Safety analyses were based on all subjects treated, including all subjects who received any dose of corifollitropin alfa. Summary statistics and/or incidence rates were provided for descriptive safety endpoints including AEs, deaths, laboratory parameters, vital signs, and body weight.

## Results

### Disposition of subjects

The flow of participants through the study is illustrated in Additional file 2: Figure S2. All subjects were white males who ranged in age from 20 to 50 years (mean: 31.5 years). The mean weight of subjects was 84.0 kg and the mean BMI was 26.4 kg/m^2^. In the 14/18 men with a hypothalamic cause of hypogonadism, 11 presented with Kallmann syndrome, 2 were diagnosed with idiopathic HH, and 1 had other causes for HH. In the 4/18 men with a pituitary cause of hypogonadism, 3 subjects were diagnosed with panhypopituitarism (1 with coincident pituitary tumor) and 1 had pituitary malformation. In the overall population, 1 subject had received previous treatment with GnRH and 7 subjects with gonadotropins.

### Testicular size

**Fig. 1:**
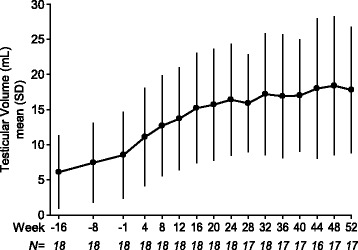
Mean (±SD) testicular volume at each study visit from pretreatment baseline to Week 52 of the combined treatment phase

Over the 52-week combined treatment phase, the mean testicular volume (±SD) increased from 8.6 (±6.09) to 17.8 (±8.93), with a geometric mean fold increase of 2.30 (95% CI: 2.03, 2.62). Starting from Week 4, the mean testicular volume increased and stabilized after Week 16 of the 52-week combined treatment phase (Fig. 1). The results for the left and right testes were consistent with that of total testicular volume (data not shown). During this period, 7/18 (39%) subjects had the twice-weekly dose of hCG uptitrated from 1500 IU to 3000 IU.

### Induction of spermatogenesis

**Fig. 2:**
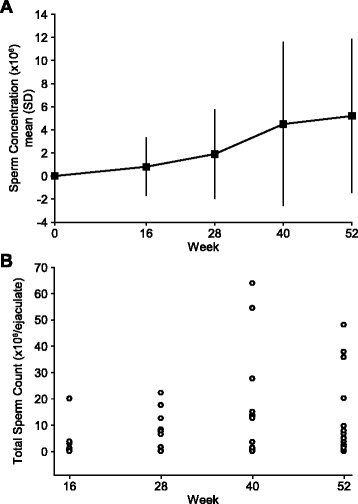
**a** Mean (±SD) sperm concentration (×10^6^) from Day 1 to Week 52 of the combined treatment phase; **b** Total sperm count (×10^6^/ejaculate) during the combined treatment phase

**Fig. 3:**
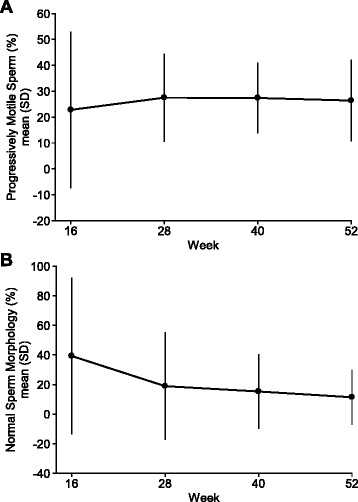
**a** Mean (±SD) percentage of progressively motile sperm during the combined treatment phase; **b** Mean (±SD) percentage of normal morphology sperm during the combined treatment phase

Fourteen (77.8%) subjects achieved a sperm count of ≥1 × 10^6^/mL at one of their assessments from the Day 1 exam at the start of corifollitropin alfa therapy in the combined treatment phase up to Week 52 (i.e.*,* the end of the combined treatment phase). The mean sperm concentration increased from 0 at Day 1 to 5.2 × 10^6^/mL at Week 52 (Fig. 2a). The total sperm count over the course of the combined treatment phase is illustrated in Fig. 2b. By the end of the combined treatment phase, an overall mean fraction (±SD) of 26.4% (±15.6%) of progressively motile sperm cells were observed, and the overall mean percentage of morphologically normal sperm cells was 11.4% (±18.5%) (Fig. 3).

### Hormonal responses

**Fig. 4:**
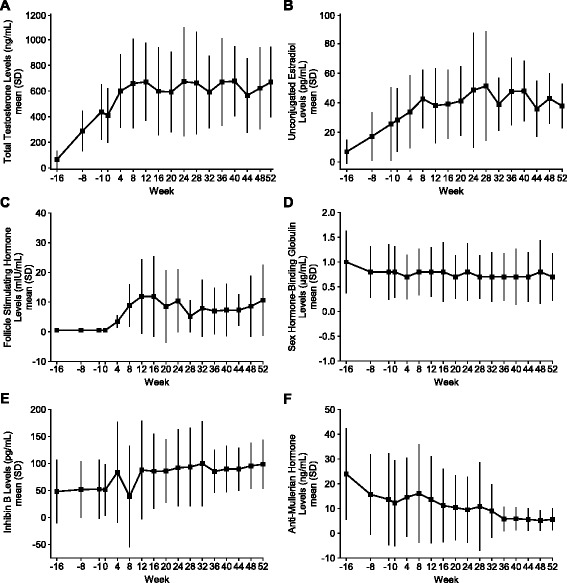
**a**–**f** Mean (±SD) hormone levels from pretreatment baseline to Week 52 of the combined treatment phase

Figure 4 illustrates the serum hormone levels from baseline to Week 52 of the combined treatment phase. During the 16-week hCG-only pretreatment phase, mean serum T levels increased from 65.2 ng/dL to 437.6 ng/dL; men who continued into the combined treatment phase had an additional mean increase of 297.8 ng/dL (from 409.7 ng/dL at Day 1 to 669.8 ng/dL at Week 52). E2 levels rose from 6.8 to 25.6 pg/mL in the pretreatment phase, with an additional mean increase of 13.4 pg/mL by Week 52 in the combined treatment phase. FSH levels did not change during the pretreatment phase of the trial, and increased from a median of 0.5 mIU/mL on Day 1 to 6.6 mIU/mL by Week 52 in the combined treatment phase. Most of this increase occurred during the first 8 weeks, after which time FSH levels remained stable to the end of the study and then decreased to near Day 1 levels (0.5 mIU/mL) by the final follow-up visit when treatment had been discontinued (≥21 days after the last dose of corifollitropin alfa and ≥7 days after the last dose of hCG). Mean serum SHBG levels remained fairly constant over the treatment period. Inhibin-B levels remained relatively stable during the hCG-only pretreatment phase, but showed a mean increase during the combined treatment phase, from 51.3 pg/mL (±46.92) on Day 1 to 98.6 pg/mL (±44.28) at Week 52 (1.9 fold increase). Mean AMH levels declined slightly during the pretreatment phase of the trial, from 24.0 to 13.7 ng/mL, and a greater drop (>2-fold) occurred during the combined treatment phase. The levels of LH did not change during either the pretreatment phase or the combined treatment phase (data not shown).

### Safety

No subject developed confirmed anti-corifollitropin alfa antibodies during this trial. A total of 11 (61.1%) subjects had one or more AEs during the combined treatment phase (Additional file 3: Table S1). The most frequently reported AEs were nasopharyngitis (22.2%), estradiol increased (16.7%), and headache (16.7%); the remaining AEs were reported in two or fewer subjects. Five (27.8%) subjects had AEs that were considered by the investigators to be drug-related. The most frequently reported drug-related AEs were estradiol increased (*n* = 3), blood testosterone increased (*n* = 2), and blood testosterone decreased (*n* = 2); there were no other AEs considered to be drug-related reported in more than one subject during the combined treatment phase. There were no SAEs and no deaths reported during the combined treatment phase. One subject was discontinued from the trial during the combined treatment phase because of an AE (blood testosterone increased to 1528 ng/dL at Week 32), which was considered by the investigator to be drug-related. No clinically relevant changes in laboratory values or vital signs were observed.

## Discussion

Combined treatment with corifollitropin alfa and hCG for 52 weeks resulted in a greater than two-fold increase in testicular volume in men with HH who remained azoospermic after 16 weeks of treatment with hCG treatment alone, meeting the primary hypothesis of the study. Treatment with corifollitropin alfa resulted in an increase in the sperm count of ≥1 × 10^6^/mL at or before week 52 of the combined treatment phase in 14 of 18 (77.8%) men, meeting the key secondary hypothesis of the study. No subject developed antibodies to corifollitropin alfa. Treatment was generally well tolerated and no new safety signals were identified during the conduct of this study.

HH is a rare disease affecting both men and women. In men, the lack of endogenous FSH and LH results in insufficient testicular hormone production and lack of spermatogenesis. The current treatment of pre- or post-pubertal HH to achieve virilization and fertility requires three-times-weekly injections of gonadotropins (2× hCG and 3× recFSH). Whereas combined treatment with hCG and recFSH has been successful for the treatment of HH [6, 13–16], the use of corifollitropin alfa in place of recFSH would reduce the number of required injections and may improve patient satisfaction and compliance. This is important, as treatment for infertility is often prolonged and may require up to two years of continuous treatment [17].

Corifollitropin alfa resulted in an increase in Total T, E2, and inhibin-B levels, and a corresponding decrease in AMH levels. These changes are consistent with what is expected prior to the onset of spermatogenesis. The increase in T was slightly less than that observed in the recFSH trial, but this did not appear to negatively impact the primary or key secondary outcomes of the trial. The FSH levels, endogenous only at baseline, endogenous plus corifollitropin alfa levels at Week 52, increased during the first 8 weeks of the combined treatment phase, remained relatively stable until Week 52, and returned to near Day 1 levels following treatment discontinuation. This pattern is consistent with the expected outcomes of this trial. All other laboratory values, except for AMH which was not measured in the recFSH trial, were similar to those observed previously with recFSH [6].

Following the changes in serum hormone levels, sperm counts increased in 14 of 18 subjects during the last half of the trial. This finding is consistent with the time required to complete spermatogenesis. Assuming an immediate response to corifollitropin alfa treatment, sperm would not be expected to appear in seminal fluid before approximately 75 days after initiation of treatment in an azoospermic male. The increase in the percent of sperm with abnormal morphology over the course of the trial may be related to the increased number of sperm available for assessment from men who were azoospermic at baseline, especially considering that the range of morphologically abnormal sperm is 52–97% in healthy, fertile men [12]. A similar increase (from 74.5–93.2%) in the percentage of sperm with abnormal morphology was observed in an earlier trial in men with HH, in which spermatogenesis was induced with hCG and recFSH [6]; thus, the present finding is not unexpected.

The increases in testicular volume and sperm counts observed in the present study suggest that corifollitropin alfa can effectively replace recFSH in the treatment regimen of adult men with HH desiring fertility. The target sperm count of at least 1 × 10^6^/mL was selected based on comparable studies showing that such concentrations can realistically be attained within the time frame of a clinical trial involving HH patients. For instance, in a previous study of recFSH and hCG for the treatment of HH, 47% of men achieved sperm counts ≥1 × 10^6^/mL [6]. In the current study, nearly 78% of men treated with corifollitropin alfa and hCG reached this threshold. The sustained elevation in serum FSH levels resulting from corifollitropin alfa injections most likely contribute to the high success rate of this regimen. Although sperm concentrations and counts remained in the low range, it should be noted that the sperm produced by these men would be expected to have a high capacity to fertilize eggs, either spontaneously or by intracytoplasmic sperm injection (ICSI), as pregnancy rates of 65–90% in previous studies have been shown [4, 14, 18]. While the chances for paternity in corifollitropin alfa-treated HH patients should be high based on data from these previous studies, it was not one of the endpoints examined in the present study.

Adverse events were reported for 11 (61.1%) subjects who received corifollitropin alfa. The investigators considered the AEs to be drug-related in 5 of these 11 subjects. The most commonly reported AEs were related to changes in serum hormone levels (increasing or decreasing T, or an increase in E2), and none was reported as an SAE. One subject was discontinued from treatment because of an increase in the Total T level to 1528 ng/dL. This level was reversible and declined after discontinuation of treatment. The event was judged by the investigator as likely to be drug-related. There were no deaths reported in the trial. These findings support the overall safety of corifollitropin alfa.

This study had several limitations. This was a single-arm study that did not employ a control group. In addition, the number of men included in this study was small. Finally, sperm morphologies were performed locally, a test that might introduce variability across laboratories; however, all sites were trained with the standardized methodology prior to the start of the study to mitigate the potential for such variability.

## Conclusions

In conclusion, in men with HH pretreated with hCG alone for 16 weeks, addition of corifollitropin alfa to ongoing hCG treatment resulted in more than a doubling in testicular volume and an increase in sperm count to at least 1 × 10^6^/mL in 14 of 18 men, with appropriate increases in total T, E2, and inhibin B, and a decrease in AMH levels. In addition, there were no new safety signals identified, with no development of anti-corifollitropin alfa antibodies. Taken together, these results suggest that long-acting corifollitropin alfa can effectively and safely replace recFSH in the treatment regimen of adult men with HH desiring fertility. Additional studies exploring the role of corifollitropin alfa in the treatment of male HH are warranted.

## Additional files

Additional file 1: Figure S1. Study design. (EPS 1160 kb)
Additional file 2: Figure S2. Subject disposition. (EPS 1138 kb)
Additional file 3: Table S1. Summary of Adverse Events in the Combined Treatment Phase. (DOCX 29 kb)

## Abbreviations

ABL: Analytical Biochemical Laboratory
AEs: Adverse events
ALT: Alanine aminotransferase
AMH: anti-Müllerian hormone
ART: Assisted reproductive technology
AST: Aspartate aminotransferase
CI: Confidence interval
E2: Estradiol
EIA: Enzyme immunoassay
FSH: Follicle-stimulating hormone
GnRH: Gonadotropin-releasing hormone
hCG: Human chorionic gonadotropin
HH: Hypogonadotropic hypogonadism
hMG: Human menopausal gonadotropin
ICSI: Intracytoplasmic sperm injection
LH: Luteinizing hormone
LLOQ: Lower limit of quantitation
PD: Pharmacodynamic
recFSH: Recombinant follicle-stimulating hormone
SAEs: Serious adverse events
SC: Subcutaneously
SD: Standard deviation
SHBG: Sex hormone-binding globulin
T: Testosterone
ULN: Upper limit of normal
