# Supplementary material for: An open-label clinical trial to investigate the efficacy and safety of corifollitropin alfa combined with hCG in adult men with hypogonadotropic hypogonadism
Source: Reprod Biol Endocrinol. 2017 Mar 7;15:17. doi: 10.1186/s12958-017-0232-y (PMC5341390; doi:10.1186/s12958-017-0232-y)
Supplement: Additional file 3: Table S1. — Summary of Adverse Events in the Combined Treatment Phase. (DOCX 29 kb) [file 12958_2017_232_MOESM3_ESM.docx]

| **Table S1** Summary of Adverse Events in the Combined Treatment Phase | |
| --- | --- |
| **Subjects** | **MK-8962 150 μg + hCG N = 18 n (%)** |
| **Overall Summary** | |
| With ≥1 AE | 11 (61.1) |
| Drug-related AEs | 5 (27.8) |
| Serious AEs | 0 (0.0) |
| Serious drug-related AEs | 0 (0.0) |
| Deaths | 0 (0.0) |
| Discontinued due to an AE | 1 (5.6) |
| Drug-related AE | 1 (5.6) |
| **Adverse Events Reported for ≥2 Subjects** | |
| With ≥1 AE | 11 (61.1) |
| Nasopharyngitis | 4 (22.2) |
| Headache | 3 (16.7) |
| Estradiol increased | 3 (16.7) |
| Blood testosterone decreased | 2 (11.1) |
| Blood testosterone increased | 2 (11.1) |
